# No Evidence of Sexual Risk Compensation in the iPrEx Trial of Daily Oral HIV Preexposure Prophylaxis

**DOI:** 10.1371/journal.pone.0081997

**Published:** 2013-12-18

**Authors:** Julia L. Marcus, David V. Glidden, Kenneth H. Mayer, Albert Y. Liu, Susan P. Buchbinder, K. Rivet Amico, Vanessa McMahan, Esper Georges Kallas, Orlando Montoya-Herrera, Jose Pilotto, Robert M. Grant

**Affiliations:** 1 Gladstone Institute of Virology and Immunology, San Francisco, California, United States of America; 2 University of California, Berkeley, California, United States of America; 3 University of California San Francisco, San Francisco, California, United States of America; 4 Fenway Institute, Fenway Health, Boston, Massachusetts, United States of America; 5 Beth Israel Deaconess Medical Center, Boston, Massachusetts, United States of America; 6 Bridge HIV, San Francisco Department of Public Health, San Francisco, California, United States of America; 7 Center for Health, Intervention and Prevention, University of Connecticut, Storrs, Connecticut, United States of America; 8 Division of Clinical Immunology and Allergy, School of Medicine, University of São Paulo, São Paulo, Brazil; 9 Fundación Ecuatoriana Equidad, Guayaquil, Guayas, Ecuador; 10 Laboratorio de AIDS e Imunologia Molecular, Hospital Geral de Nova Iguacu, Rio de Janeiro, Brazil; Rollins School of Public Health, United States of America

## Abstract

**Objective:**

Preexposure prophylaxis (PrEP) with emtricitabine/tenofovir disoproxil fumarate (FTC/TDF) reduced HIV acquisition in the iPrEx trial among men who have sex with men and transgender women. Self-reported sexual risk behavior decreased overall, but may be affected by reporting bias. We evaluated potential risk compensation using biomarkers of sexual risk behavior.

**Design and methods:**

Sexual practices were assessed at baseline and quarterly thereafter; perceived treatment assignment and PrEP efficacy beliefs were assessed at 12 weeks. Among participants with ≥1 follow-up behavioral assessment, sexual behavior, syphilis, and HIV infection were compared by perceived treatment assignment, actual treatment assignment, and perceived PrEP efficacy.

**Results:**

Overall, acute HIV infection and syphilis decreased during follow-up. Compared with participants believing they were receiving placebo, participants believing they were receiving FTC/TDF reported more receptive anal intercourse partners prior to initiating drug (12.8 vs. 7.7, *P* = 0.04). Belief in receiving FTC/TDF was not associated with an increase in receptive anal intercourse with no condom (ncRAI) from baseline through follow-up (risk ratio [RR] 0.9, 95% confidence interval [CI]: 0.6–1.4; *P* = 0.75), nor with a decrease after stopping study drug (RR 0.8, 95% CI: 0.5–1.3; *P* = 0.46). In the placebo arm, there were trends toward lower HIV incidence among participants believing they were receiving FTC/TDF (incidence rate ratio [IRR] 0.8, 95% CI: 0.4–1.8; *P* = 0.26) and also believing it was highly effective (IRR 0.5, 95% CI: 0.1–1.7; *P* = 0.12).

**Conclusions:**

There was no evidence of sexual risk compensation in iPrEx. Participants believing they were receiving FTC/TDF had more partners prior to initiating drug, suggesting that risk behavior was not a consequence of PrEP use.

## Introduction

Despite decades of prevention efforts, human immunodeficiency virus (HIV) infection is a global pandemic, with 2.5 million people newly infected in 2011 [1] and men who have sex with men (MSM) disproportionately affected worldwide. [2], [3] As behavioral interventions have not been sufficient to end the epidemic, research has also included biomedical interventions. [4] In the primary analysis of the iPrEx randomized controlled trial (RCT), preexposure prophylaxis (PrEP) with once-daily oral emtricitabine/tenofovir disoproxil fumarate (FTC/TDF) reduced the risk of HIV acquisition by 44% among HIV-uninfected MSM and transgender women compared with placebo, and by 92% among participants with detectable drug levels; a subsequent modeling study estimated a 96–99% risk reduction among those with drug concentrations commensurate with daily dosing. [5], [6] Although two interventions in African women – the oral and topical tenofovir arms of the VOICE study and the FEM-PrEP [7] trial of FTC/TDF – were closed early for futility, and the FTC/TDF arm of the VOICE study later showed no effect, [8] the Partners PrEP study in heterosexual serodiscordant couples [9] and the CDC PrEP trial in heterosexual men and women [10] found oral FTC/TDF to be highly effective.

Like other prevention strategies – including circumcision, condom use, and HIV testing – excitement about PrEP has been tempered by concerns about potential increases in sexual risk behavior among users, [11], [12] an effect defined as “risk compensation.” [13] According to risk compensation theory, individuals adjust their behavior in response to changes in their perceived level of risk. [13], [14] This theory has been used as an explanation for why population-level benefits of seatbelt use have not been observed, [15] sunscreen use has been associated with an increased risk of melanoma, [16] and condom promotion has had a limited population-level impact on HIV in communities with generalized epidemics. [15] Risk compensation has also been linked to increases in sexual risk behavior coinciding with the introduction of combination antiretroviral therapy, an effect called “treatment optimism.” [17] Similarly, PrEP optimism could result in increased risk behavior that could potentially reduce its protection against HIV acquisition. [18], [19].

Empirically, previous studies of biomedical HIV-prevention interventions have found mixed evidence of risk compensation. One small HIV vaccine trial reported an overall increase in insertive anal intercourse with no condom, but no change in receptive anal intercourse, the strongest risk factor for HIV acquisition. [20] A trial of male circumcision found a higher number of sexual partners and contacts in the circumcised group compared with the control group, [21] although risk behavior in both groups was lower during the study than at baseline. There was no difference in risk behaviors between study arms in another two trials of male circumcision, [22], [23] with one of those studies finding no evidence of risk compensation during three years of post-trial follow-up. [24] There was no increase in risk behaviors in an HIV vaccine efficacy trial, [25] and overall decreases in risk behavior in two longitudinal studies of post-exposure prophylaxis (PEP). [26], [27] Likewise, there has been no suggestion of risk compensation among PrEP trial participants to date; in fact, decreases in risk behavior have been observed. [5], [7], [9], [10], [28], [29] However, only two PrEP trials explored changes in sexual risk behavior in subgroups of participants, [29], [30] and none specifically assessed risk compensation using biomarkers of sexual risk behavior.

Although an overall decrease in sexual risk behavior was observed among participants in iPrEx, [5] self-reported behavior can be biased by social desirability. Furthermore, behavior in the trial context may differ from behavior during subsequent implementation, when there is no longer the possibility that the user is receiving a placebo. To address these concerns, we conducted an analysis using biomarkers of sexual risk behavior and focusing on the group of participants who believed they were receiving the active drug and that it was effective. We aimed to describe trends in sexual behavior during study follow-up and after stopping study drug; to describe trends in the acquisition of HIV and syphilis; and to determine whether participants who believed they were receiving FTC/TDF and that it was highly effective had increased sexual risk behavior or increased risk of HIV or syphilis acquisition compared with participants who believed they were receiving placebo.

## Methods

### Ethics Statement

The iPrEx study was approved by the Committee on Human Research at the University of California, San Francisco, as well as local institutional review boards at each study site: Comité Institucional de Bioética, Asociación Civil Impacta Salud y Educación, Lima, Peru; Universidad San Francisco de Quito, IRB #1, Quito, Ecuador; Fenway Community Health Institutional Review Board, Boston, MA; Comissão de Ética para Análise de Projetos de Pesquisa, CAPPesq Hospital das Clínicas da Faculdade de Medicina da USP, São Paulo, Brazil; Comitê de Ética em Pesquisa, Hospital Universitario Clementino Fraga Filho/Universidade Federal de Rio de Janeiro, Rio de Janeiro, Brazil; Comitê de Ética em Pesquisa do Instituto de Pesquisa Clínica Evandro Chagas, Rio de Janeiro, Brazil; National IRB: Comissão Nacional de Ética em Pesquisa – CONEP, Ministério da Saúde, Brasília, Brazil; University of Cape Town Research Ethics Committee, Cape Town, South Africa; Human Experimentation Committee, Research Institute for Health Sciences, Chiang Mai, Thailand; Ethical Review Committee for Research in Human Subjects, Department of Medical Services, Ministry of Public Health, Nonthaburi, Thailand; Research Ethics Committee, Faculty of Medicine, Chiang Mai University, Chiang Mai, Thailand. Written informed consent was obtained from each participant prior to enrollment in the study.

### Study Population

A detailed description of the iPrEx study has been previously published. [5] Briefly, the study enrolled 2,499 MSM and transgender women at risk for HIV infection at 11 sites in Peru, Ecuador, South Africa, Brazil, Thailand, and the United States during 2007–2009. At the enrollment visit, participants were randomized to receive FTC/TDF or placebo and were instructed to take one tablet daily. Monthly visits included risk-reduction and adherence counseling. Serological testing for syphilis was performed at screening and every six months during follow-up. Symptoms of depression were measured semiannually beginning in July of 2009, defined as a score of ≥16 on the Centers for Epidemiologic Studies Depression Scale (CESD); data from the first assessment were included in the current analysis. The primary analysis included visits through the pre-specified cutoff date of May 1, 2010, whereas the current analysis includes follow-up visits through November 21, 2010. Study results were not released until December, 2010.

### Sexual Behavior

Sexual practices during the previous three months were assessed by interviewer-administered questionnaires at screening and at quarterly visits subsequent to the enrollment visit, which occurred within 28 days of screening. We initially assessed trends in the number of receptive anal intercourse (RAI) partners and the proportion of those partners with whom a condom was consistently used. For the remainder of the analysis, we focused on any RAI with no condom (ncRAI), a dichotomized variable that is a composite of the first two outcomes and the behavior most strongly associated with HIV acquisition in iPrEx. [5] For the current analysis, baseline sexual practices refer to behavior reported at the screening visit, while sexual practices during follow-up refer to behavior reported at any visits subsequent to enrollment.

### Perceived Treatment Assignment and PrEP Efficacy Beliefs

At quarterly visits, participants were asked to which treatment group they believed they had been assigned. Response options were “I don’t know” or “I strongly [or somewhat] think I am in the Truvada [or placebo] group.” The “strongly” and “somewhat” categories were collapsed for analysis. Unless otherwise specified, we used data on perceived treatment group collected at the first quarterly visit after enrollment (12-week visit). Perceived treatment group was evenly distributed by actual treatment group at the 12-week visit. [5] At the same visit, participants were asked about how good they thought FTC/TDF was at preventing HIV infection on a scale from 1 (doesn’t prevent) to 10 (prevents all the time). This variable was collapsed into high (6–10), low (1–5), and “don’t know.”

### Statistical Analysis

To assess trends in sexual behavior, we calculated the mean number of RAI partners and the proportion of those partners using a condom, using Poisson regression to estimate change over time and t-tests to compare means by perceived treatment group. We assessed the relationship between beliefs in treatment assignment and PrEP efficacy, in addition to other participant characteristics, and changes in reported ncRAI. We assessed such changes in sexual behavior in two separate analyses: from baseline to any time during follow-up, and from the visit at which study drug was discontinued (stop visit) to the visit eight weeks later (post-stop visit). For the latter analysis, perceived treatment group was extracted from data collected at the stop visit or the closest prior measurement. We used mixed log-binomial models to estimate risk ratios (RR), with a random effect for study site to account for unmeasured differences across sites. Participants were excluded if they did not have at least one quarterly assessment during which data on sexual behavior were collected.

To measure trends in sexual behavior using objective indices, we used retrospectively tested plasma specimens to calculate the baseline prevalence of acute HIV infection as an estimate of HIV incidence in the short period prior to enrollment; we then compared baseline prevalence to the prevalence of acute HIV infection during follow-up in each arm of the study. Acute infection was defined as plasma HIV RNA positive and HIV-antibody negative at baseline, and as the first HIV RNA positive while HIV-antibody negative during follow-up. We also calculated syphilis incidence in each treatment arm and by perceived treatment group among participants without seroreactivity for syphilis at baseline, with incidence calculated for each one-year time period and using chi-square tests for trend. Finally, we used Poisson regression to estimate the association between HIV incidence and beliefs in treatment assignment and PrEP efficacy among participants in the placebo arm.

All analyses were conducted in Stata 12 and SAS 9.2.

## Results

### Study Participants

Of the 2,499 participants enrolled in iPrEx, 2,408 (96.4%) completed at least one follow-up quarterly assessment and were included in analyses of trends in sexual behavior. All participants were born male, and 313 (13.0%) identified as women or transgender. At enrollment, the mean age was 25 years (range 18–67). Loss to follow-up was less frequent among participants reporting ncRAI at baseline (hazard ratio [HR] 0.8, 95% confidence interval [CI]: 0.7–1.0; *P* = 0.02) and among participants reporting diagnosis of a sexually transmitted infection (STI) in the six months prior to screening or having an STI symptom at screening (HR 0.8, 95% CI: 0.6–1.0; *P* = 0.06).

### Trends in HIV and Syphilis by Actual Treatment Group

At baseline, the prevalence of acute HIV infection was 0.4% overall (10/2,499). During follow-up, acute infection decreased 3.8-fold (95% CI: 1.5–9.5; *P* = 0.004) to 0.1% among participants in the placebo arm and 6.5-fold (95% CI: 2.2–20.2; *P* = 0.002) to 0.06% in the active arm. Syphilis incidence also declined in both treatment arms during follow-up (*P*<0.001; Table 1).

**Table 1:** Syphilis incidence by treatment group among participants testing seronegative at baseline.*

	FTC/TDF			Placebo	
Visit year	Incidence (person-years)	Incidence rate		Incidence (person-years)	Incidence rate
1	60 (946)	6.3		54 (971)	5.6
2	20 (541)	3.7		28 (569)	4.9
3	7 (178)	3.9		7 (179)	3.9
4	0 (3)	0.0		0 (3)	0.0
	*P* trend <0.001			*P* trend <0.001	

Incidences exclude recurrent syphilis.

### Perceived Treatment Group and PrEP Efficacy Beliefs

At the 12-week visit, 553 participants (25.1%) believed they were in the FTC/TDF group, 223 (10.1%) believed they were in the placebo group, and 1,429 (64.8%) reported that they did not know their treatment assignment (Table 2). At the stop visit, the number of participants reporting that they did not know their treatment assignment increased to 1,631 (71.7%), with 471 participants (20.7%) believing they were in the FTC/TDF group and 174 (7.6%) believing they were in the placebo group; 71.5% of participants reported consistent perceptions of their treatment assignment across the visits. The proportion of participants believing PrEP was highly effective increased from 23.5% at the 12-week visit to 26.8% at the stop visit (*P*<0.001).

**Table 2:** Baseline characteristics by perceived treatment group.*

	Perceived FTC/TDF n = 553	Perceived placebo n = 223	Don’t know n = 1429	TotalN = 2205	*P*-value
Age group, years – n (%)					0.43
18–24	263 (48)	111 (50)	726 (51)	1100 (50)	
≥25	290 (52)	112 (50)	703 (49)	1105 (50)	
Education – n (%)					0.29
Less than secondary	122 (22)	38 (17)	292 (21)	452 (21)	
Completed secondary	423 (78)	181 (83)	1125 (79)	1729 (79)	
Transgender or female sexual identity − n (%)					0.21
Yes	72 (13)	20 (9)	188 (13)	280 (13)	
No	481 (87)	203 (91)	1241 (87)	1925 (87)	
Previously tested for HIV – n (%)					0.03
Yes	427 (78)	178 (81)	1051 (74)	1656 (76)	
No	120 (22)	43 (19)	370 (26)	533 (24)	
Number of RAI partners at baseline – mean (SD)	13 (36)	8 (16)	12 (28)	12 (29)	0.12
Percent of RAI partners using a condom at baseline – mean (SD)	55 (37)	51 (37)	49 (37)	50 (37)	0.02
ncRAI at baseline – n (%)					0.28
Yes	317 (57)	135 (61)	875 (61)	1327 (60)	
No	236 (43)	88 (39)	554 (39)	878 (40)	
No. of alcoholic drinks on days when participant drank inpast month at baseline – n (%)					<0.001
0–4	283 (52)	109 (50)	576 (41)	968 (45)	
≥5	259 (48)	109 (50)	813 (59)	1181 (55)	
Cocaine or crack use in past month at baseline – n (%)					0.09
Yes	41 (8)	16 (7)	73 (5)	130 (6)	
No	505 (92)	205 (93)	1352 (95)	2062 (94)	
Symptoms of depression – n (%)					0.62
Yes	106 (22)	45 (23)	304 (24)	455 (23)	
No	386 (78)	151 (77)	977 (76)	1514 (77)	
Perceived PrEP effectiveness at 12 weeks – n (%)					<0.001
High	177 (33)	57 (26)	269 (20)	503 (24)	
Low	164 (31)	67 (31)	330 (24)	561 (26)	
Don’t know	194 (36)	93 (43)	778 (57)	1065 (50)	

= receptive anal intercourse with no condom; SD = standard deviation. Excludes participants missing data on perceived treatment group at the first quarterly visit subsequent to enrollment (n = 203). Ns may not add up to column totals because of missing data on participant characteristics. *P*-values by chi-square or analysis of variance. ncRAI

### Change in Sexual Behavior from Baseline through Follow-up

**Figure 1:**
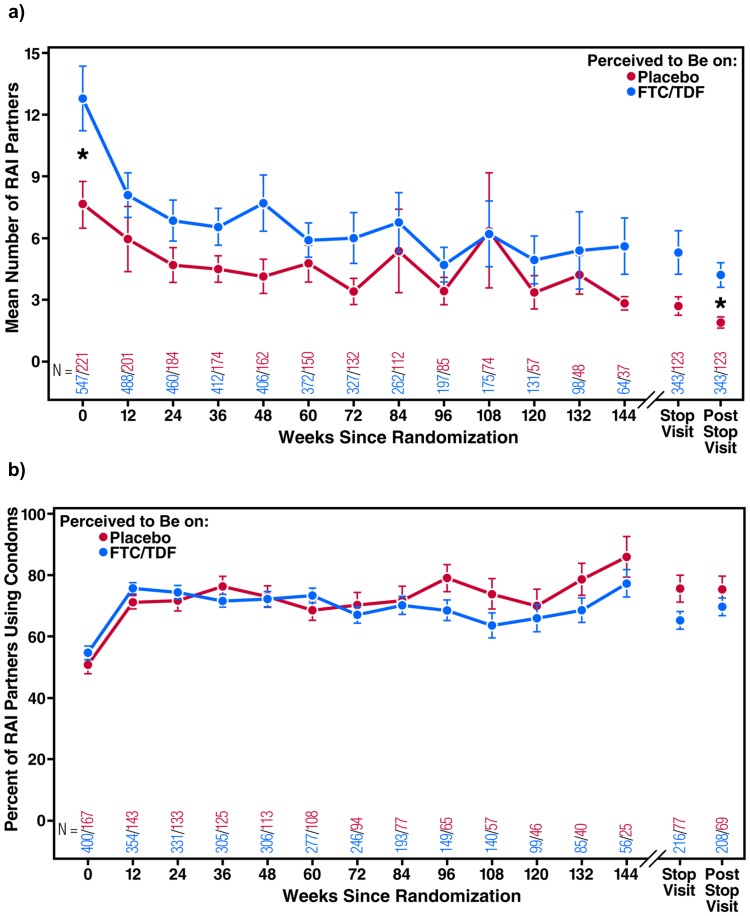
Sexual behavior by perceived treatment group. Figure 1a shows the mean number of receptive anal intercourse (RAI) partners in the past 3 months by perceived treatment group at 12 weeks. Figure 1b shows the percent of those partners using a condom by perceived treatment group at 12 weeks. Asterisks indicate *P*<0.05 by t-test.

The mean number of RAI partners decreased (*P*<0.001), and the proportion of those partners using a condom increased (*P*<0.001), from baseline through follow-up in both participants who believed they were receiving FTC/TDF and those who believed they were receiving placebo (Figure 1a–b). Participants who believed they were receiving FTC/TDF had a higher mean number of RAI partners in the three months prior to baseline compared to those who believed they were receiving placebo (12.8 vs. 7.7; *P* = 0.04). There were no significant differences in the proportion of partners using a condom by perceived treatment group. Overall, 1,108 participants (46.0%) reported ncRAI both at baseline and at least once during follow-up, 731 (30.4%) reported no ncRAI at baseline nor during follow-up, 331 (13.7%) reported ncRAI at baseline but not during follow-up, and 238 (9.9%) reported ncRAI at least once during follow-up but not at baseline. Of the 1,439 participants (57.6%) who reported ncRAI at baseline, 331 (23.0%) did not report ncRAI again during follow-up. Among those reporting ncRAI at baseline, those who had never previously tested for HIV were more likely to have a decrease in ncRAI during follow-up (RR 1.4, 95% CI: 1.1–1.7; *P*<0.01; Table 3). A decrease in ncRAI was less likely among participants who identified as women or transgender (RR 0.8, 95% CI: 0.6–1.0; *P* = 0.04), were less than 25 years of age (RR 0.8, 95% CI: 0.7–1.0; *P* = 0.04), or reported symptoms of depression (RR 0.7, 95% CI: 0.6–1.0; *P* = 0.03).

**Table 3:** Participant characteristics associated with a change in sexual behavior from baseline through follow-up.*

	Participants with no ncRAI at baseline (n = 969)		Participants with ncRAI at baseline (n = 1439)	
	ncRAI during follow-upRR (95% CI)	P-value	No ncRAI during follow-upRR (95% CI)	P-value
Age <25 (ref. ≥25)	1.3 (1.0, 1.6)	0.03	0.8 (0.7, 1.0)	0.04
Completed secondary school	1.1 (0.8, 1.5)	0.54	0.9 (0.7, 1.2)	0.61
Transgender or female sexual identity	1.7 (1.2, 2.5)	0.002	0.8 (0.6, 1.0)	0.04
≥5 drinks on drinking days	1.0 (0.8, 1.2)	0.78	1.0 (0.8, 1.2)	0.89
Cocaine or crack use	1.0 (0.7, 1.5)	0.95	1.0 (0.7, 1.5)	0.98
Never previously tested for HIV	0.7 (0.5, 0.9)	0.01	1.4 (1.1, 1.7)	0.004
Symptoms of depression	1.6 (1.2, 2.1)	0.002	0.7 (0.6, 1.0)	0.03
Perceived treatment group FTC/TDF (ref. placebo)	0.9 (0.6, 1.4)	0.75	0.8 (0.6, 1.2)	0.40
Perceived high PrEP effectiveness (ref. low)	0.7 (0.5, 1.0)	0.06	1.2 (0.9, 1.7)	0.20
Perceived group FTC/TDF and perceived high PrEPeffectiveness (ref. perceived placebo)	0.8 (0.3, 2.1)	0.68	0.7 (0.4, 1.3)	0.25

= receptive anal intercourse with no condom; RR = risk ratio; CI = confidence interval. By mixed log-binomial regression models with study site as a random effect. ncRAI

Of the 969 (38.8%) participants who did not report ncRAI at baseline, 238 (24.6%) reported ncRAI at least once during follow-up. Among participants who did not report ncRAI at baseline, an increase in ncRAI during follow-up was associated with identifying as a woman or transgender (RR 1.7, 95% CI: 1.2–2.5; *P* = 0.002), being less than 25 years of age (RR 1.3, 95% CI: 1.0–1.6; *P* = 0.03), or reporting symptoms of depression (RR 1.6, 95% CI: 1.2–2.1; *P* = 0.002). An increase in ncRAI during follow-up was less likely among those who had not previously tested for HIV (RR 0.7, 95% CI: 0.5–0.9; *P* = 0.01). Among participants reporting no ncRAI at baseline, participants who believed they were receiving FTC/TDF were no more likely to report ncRAI during follow-up compared with participants who believed they were receiving placebo (RR 0.9, 95% CI: 0.6–1.4; *P* = 0.75), with similar results among those who also believed PrEP was highly effective (RR 0.8, 95% CI: 0.3–2.1; *P* = 0.68).

### Change in Sexual Behavior after Stopping Study Drug

Of the 1,743 participants with sexual behavior assessments both at the time of stopping drug and eight weeks later, 461 (26.4%) reported ncRAI at the stop visit and 393 (22.5%) reported ncRAI at the post-stop visit, representing a decrease of 3.9% (95% CI: 2.0–5.8) in reported ncRAI after stopping study drug (*P*<0.001 by paired t-test). Of the 461 participants who reported ncRAI at the stop visit, 182 (39.5%) reported no ncRAI at the post-stop visit. Participants who believed they were receiving FTC/TDF were no more likely to decrease ncRAI after stopping study drug compared with participants who believed they were receiving placebo (RR 0.8, 95% CI: 0.5–1.3; *P* = 0.46), with similar results among participants who also believed PrEP was highly effective (RR 0.8, 95% CI: 0.3–2.2; *P* = 0.63).

### Trends in HIV and Syphilis by Perceived Treatment Group

There was no difference in overall syphilis incidence during follow-up by perceived treatment group (*P* = 0.80). Among participants in the placebo arm, HIV incidence was not significantly higher among participants who believed they were receiving FTC/TDF compared with participants who believed they were receiving placebo (IRR 0.8, 95% CI: 0.4–1.8; *P* = 0.26). There was also no difference in HIV incidence between participants who believed FTC/TDF was highly effective compared with participants who believed FTC/TDF was less effective (IRR 1.2, 95% CI: 0.6–2.5; *P* = 0.26). Compared with participants who believed they were receiving placebo, those who both believed they were receiving FTC/TDF and that it was highly effective were not at increased risk of acquiring HIV during follow-up (IRR 0.5, 95% CI: 0.1–1.7: *P* = 0.12).

## Discussion

In this study of participants in a trial of daily oral FTC/TDF PrEP, we found no evidence of risk compensation, a finding that is largely consistent with other HIV-prevention studies of PrEP, [7], [9], [10], [28], [29], [30] male circumcision, [22], [23], [24] vaccines, [25] and PEP. [26], [27] There was an overall trend toward safer sexual behavior, which was supported by decreases in syphilis and HIV infection. Participants who believed they were receiving FTC/TDF had higher numbers of receptive partners at baseline, prior to initiating study drug, suggesting that risk behavior was not a consequence of receiving PrEP. The small decrease in the proportion of participants reporting ncRAI after stopping study drug was consistent with the overall time trends in RAI partners and condom use, and did not differ by perceived treatment group. Our results do not support the concerns arising from risk compensation theory: that participants who believed they were receiving FTC/TDF and that it was effective would be more likely to increase their risk behavior while on study drug or decrease their risk behavior after stopping it.

Of note, the number of RAI partners was significantly higher in the three months prior to baseline among participants who later reported believing they were assigned to FTC/TDF. Cross-sectional studies have found that optimism about the benefits of combination antiretroviral therapy was associated with riskier sexual behavior, [17] but were not able to determine causality – i.e., whether optimism about treatment made individuals feel comfortable taking more risk, or whether individuals having riskier sex were more likely to be optimistic about therapy. Our results suggest that belief in treatment assignment may have been a consequence of sexual practices, rather than sexual practices being a consequence of receiving PrEP. [31] For participants who thought they were receiving FTC/TDF, that belief could have served as a psychological coping mechanism to reduce concerns about their sexual behavior, thus minimizing cognitive dissonance. [32].

One-quarter of participants had never been tested for HIV prior to their iPrEx screening visit and reported safer sexual behavior during study follow-up than those who had previously tested. Prior studies conducted among MSM [33] and adults in developing countries [34] have found decreases in sexual risk behavior associated with HIV testing. That effect has been more pronounced among individuals testing positive, but the benefit of testing may be increased for HIV-negative individuals who are testing periodically, as would occur in PrEP programs. The regular testing that accompanies PrEP use, as well as the act of taking PrEP itself, could increase contemplation of HIV risk as manageable rather than inevitable and motivate other risk-reduction strategies. [35], [36], [37] Indeed, PrEP may increase self-efficacy regarding condom use and reduce fatalism about HIV by providing a daily opportunity for users to manage their own risk.

We identified participant characteristics that were associated with reporting ncRAI during follow-up after not reporting ncRAI at baseline, including symptoms of depression, younger age, and identifying as a woman or transgender. Our results are consistent with the high burden of HIV infection among male-to-female transgender individuals [38] and young MSM, [39] as well as prior studies that have found a correlation between depression and condomless sex. [40] These groups may be in particular need of attention in HIV-prevention interventions, including PrEP.

There are several limitations to our study. First, data collected during an RCT may not be generalizable to a non-experimental context; efforts to minimize optimism about the study drug and maximize condom use could have contributed to the trend toward safer sexual behavior during iPrEx. However, counseling, testing, and condom provision will accompany PrEP implementation, suggesting that a similar trend may occur among PrEP users outside of the RCT context. Furthermore, we did not see risk compensation among those whose thought they were in the active arm and that FTC/TDF was effective, a subgroup that better represents individuals using open-label PrEP. A second limitation is the use of self-reported data on sexual practices, which could have been affected by social desirability bias. However, the decreases in syphilis and HIV infection are consistent with the finding based on self-report that sexual risk behavior decreased during follow-up. Third, analyses among the group who both believed they were receiving FTC/TDF and that it was highly effective, as well as analyses of HIV incidence by perceived treatment group in the placebo arm, were limited in statistical power, resulting in wide CIs. Fourth, adherence to PrEP could have affected our analyses of HIV infection; however, this would only have impacted participants assigned to the active arm, and we also observed a decrease in HIV incidence among participants in the placebo arm. Fifth, an overall trend toward safer behavior could result from regression toward the mean. Finally, a trend toward safer behavior could also result from loss to follow-up among participants with sexual risk behavior, but participants with higher risk at baseline were more likely to be retained throughout the study.

In this study, we found no evidence of risk compensation that would offset the benefits of PrEP. Indeed, participation in the study was associated with safer sexual behavior; frequent clinic visits, HIV testing and counseling, and daily PrEP use itself may motivate and popularize safer sexual practices. Social interactions may be more important determinants of sexual decisions than individual weighing of risks and benefits, as posited by risk compensation theory, and may underlie the trends toward safety that have been observed in biomedical HIV-prevention trials.
